# End to end comparison of surface‐guided imaging versus stereoscopic X‐rays for the SRS treatment of multiple metastases with a single isocenter using 3D anthropomorphic gel phantoms

**DOI:** 10.1002/acm2.13576

**Published:** 2022-03-24

**Authors:** Victoria Bry, Daniel Saenz, Evangelos Pappas, Georgios Kalaitzakis, Nikos Papanikolaou, Karl Rasmussen

**Affiliations:** ^1^ Department of Radiation Oncology The University of Texas Health at San Antonio San Antonio Texas USA; ^2^ Department of Biomedical Sciences Radiology and Radiotherapy Sector University of West Attica Athens Greece; ^3^ Department of Medical Physics University of Crete Heraklion Greece

**Keywords:** C‐RAD Catalyst HD, end to end, immobilization, open face masks, Quality Assurance, SGRT, SRS, stereotactic radiosurgery, surface imaging

## Abstract

**Introduction:**

Two end‐to‐end tests evaluate the accuracy of a surface‐guided radiation therapy (SGRT) system (CRAD Catalyst HD) for position verification in comparison to a stereoscopic x‐ray imaging system (Brainlab Exactrac ) for single‐isocenter, multiple metastases stereotactic radiosurgery (SRS) using 3D polymer gel inserts.

**Materials and methods:**

A 3D‐printed phantom (Prime phantom, RTsafe PC, Athens, Greece) with two separate cylindrical polymer gel inserts were immobilized in open‐face masks and treated with a single isocentric, multitarget SRS plan. Planning was done in Brainlab (Elements) to treat five metastatic lesions in one fraction, and initial setup was done using cone beam computed tomography. Positional verification was done using orthogonal X‐ray imaging (Brainlab Exactrac) and/or a surface imaging system (CRAD Catalyst HD, Uppsala, Sweden), and shift discrepancies were recorded for each couch angle. Forty‐two hours after irradiation, the gel phantom was scanned in a 1.5 Tesla MRI, and images were fused with the patient computed tomography data/structure set for further analysis of spatial dose distribution.

**Results:**

Discrepancies between the CRAD Catalyst HD system and Brainlab Exactrac were <1 mm in the translational direction and <0.5° in the angular direction at noncoplanar couch angles. Dose parameters (*D*_Mean%_ **_,_** *D*_95%_) and 3D gamma index passing rates were evaluated for both setup modalities for each planned target volume (PTV) at a variety of thresholds: 3%/2 mm (Exactrac≥93.1% and CRAD ≥87.2%), 5%/2 mm (Exactrac≥95.6% and CRAD ≥94.6%), and 5%/1 mm (Exactrac≥81.8% and CRAD ≥83.7%).

**Conclusion:**

Dose metrics for a setup with surface imaging was found to be consistent with setup using x‐ray imaging, demonstrating high accuracy and reproducibility for treatment delivery. Results indicate the feasibility of using surface imaging for position verification at noncoplanar couch angles for single‐isocenter, multiple‐target SRS using end‐to‐end quality assurance (QA) testing with 3D polymer gel dosimetry.

## 1 INTRODUCTION

Brain metastasis, commonly sourced in regions of the lung, breast, or skin, occur in 20%–40% of cancer patients.^1^ Prognosis can be poor if patients are not treated,^2^ and they may experience cognitive impairments varying from headaches and focal weakness to behavioral changes, seizures, difficulty speaking.^3^ Prevalence has increased with more sensitive imaging techniques that improve detection of cancers^4^ and treatments that prolong life expectancy.^5^ Therapies for brain metastases vary from symptomatic measures, such as corticosteroids to reduce cerebral edema, to those that eradicate malignancy such as surgery, radiosurgery, or conventional radiotherapy.^1^ Whole brain radiation therapy (WBRT) has been the conventional treatment for brain metastases to provide symptomatic relief and potentially improve survival.^6^ Patients with good performance status and controlled extracranial disease can be considered for the more aggressive technique, stereotactic radiosurgery (SRS).^6^ Studies suggest that SRS alone may be preferable, rather than SRS and WBRT, as it can result in less cognitive deterioration with greater normal brain tissue sparing.^7^, ^8^, ^9^ An additional study demonstrated the benefit of SRS alone for multiple brain metastases when patients with 5–10 brain tumors showed an overall posttreatment survival similar to those treated for only 2–4.^10^ The margin of error for SRS is much lower than conventional radiotherapy^10^; however its clinical delivery requires the highest accuracy achievable to avoid complications.^11^

Achieving high accuracy required for complex SRS treatments does present multiple clinical challenges. Patient setup error, or the difference between actual and planned positioning of a patient with respect to the treatment beams during irradiation,^12^ can cause deviation of delivered dose distributions and deteriorate both patient safety and treatment efficiency. Setup error can be due to internal organ motion or by motion of patient skin with respect to their internal anatomy, thus limiting the reproducibility of patient setup using computed tomography (CT).^13^ This can be more pronounced in the cranio‐caudal direction due to spacing between consecutive image slices.^14^ Research recommends minimizing uncertainty in treatment setup errors to provide the most dosimetrically accurate treatment^15^ and provide efficient implementation of SRS techniques.

Challenges also lie within the design of the treatment plan itself. Conventional SRS treatment planning utilizes multiple isocenters for treatment, aligning each isocenter around an individual target lesion in the brain. This method is inefficient when treating multiple metastases because treatment duration is proportional to the number of lesions treated. For example, treatment duration for a single lesion may last 20 min and exceed an hour for multiple lesions.^16^ Longer treatment times can become counterproductive in that uncomfortable patients may move or shift during treatment thus further extending the duration of the treatment and may require additional imaging or larger treatment margins.^17^ To create a more efficient delivery in a realistic time frame, clinics have employed the single isocenter SRS treatment technique as studies suggest its delivery is equivalent and more efficient to multiple isocenter techniques.^18^

With advancements in immobilization techniques, SRS treatments have shifted from the exclusive method of invasive frame‐based treatments to frameless, moldable masks. Frameless SRS treatments includes the enclosed method, which force a patient to keep their eyes and mouth closed throughout the treatment process^19^ and the open‐face mask style that exposes the patient's face with freedom to move eyes and mouth. Positional verification for enclosed frameless immobilization utilizes orthogonal X‐ray image verification for couch kicks.^20^, ^21^ Utilizing the open‐face style mask allows for implementation of surface‐guided radiation therapy (SGRT) to assist in patient positioning at set up^22^ and motion management during treatment delivery.^23^, ^24^, ^25^ SGRT is an optical imaging system that informs clinicians about body positioning by tracking the surface of the patient.^17^ Clinical SGRT studies have highlighted the benefit of continuous localization technologies to account for motion uncertainty or reduce larger margins in the treatment of benign conditions.^17^ Phantom measurements suggest that SGRT is a feasible, reproducible method for intrafraction motion management for radiosurgery localization,^26^ and its use has demonstrated clinical outcomes comparable to those with conventional frame‐based frameless SRS techniques while providing greater patient comfort and faster treatment duration.^27^

In this study, we performed two end‐to‐end (E2E) Quality Assurance (QA) tests to verify the positioning accuracy of an SGRT system compared to X‐ray imaging. The E2E tests consisted of delivering a multi target, mono‐isocentric SRS treatment plan using the Varian Novalis Tx (Varian Medical Systems, Palo Alto, CA and BrainLAB, Feldkirchen, Germany), specially designed for small SRS targets with the HD‐120 MLC (High‐Definition Multileaf Collimator), which is a combination of the classic Novalis (BrainLAB, Feldkirchen, Germany) and the Trilogy (Varian Medical System, Palo Alto, CA).^28^ Phantoms equipped with 3D polymer gel dosimetry inserts (Prime phantom, RTsafe PC, Athens, Greece) were irradiated and used to model dose distributions resulting from the planned treatment.

## 2 MATERIALS AND METHODS

### 2.1 Equipment

**FIGURE 1:**
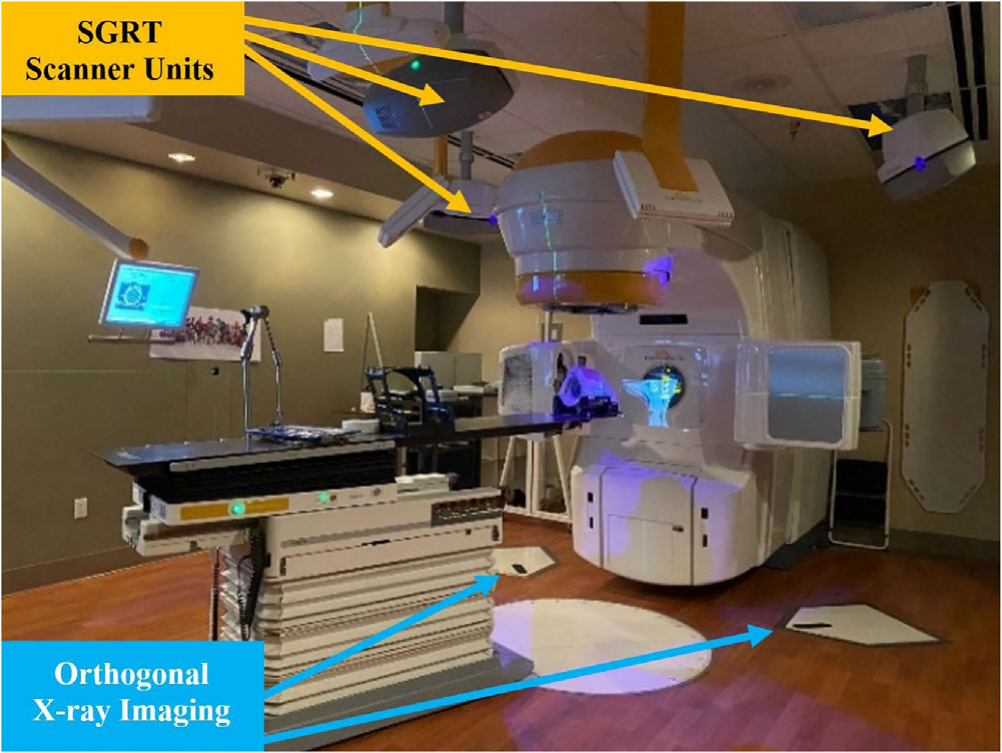
Our treatment room features the Varian Novalis Tx (Varian Medical Systems, Palo Alto, CA) with surface guided imaging scanner units (CRAD Catalyst HD, Uppsala, Sweden) positioned in the radiation treatment room above the treatment couch and the Brainlab Exactrac (Feldkirchen, Germany) Imaging system positioned with x‐ray generators in the floor.

As seen in Figure 1, our clinical SGRT system (C‐RAD Catalyst HD, Uppsala, Sweden) consists of three scanner units positioned equidistant on the ceiling of the treatment room above a linear accelerator (Varian Novalis TX, Palo Alto, CA) and undergoes monthly and daily calibrations. SGRT informs clinicians about body posture and positioning by mapping many arbitrary points on the surface of a patient over time to provide a 3D surface of the patient surface.^17^ SGRT systems can incorporate different resolutions based on the treatment site to adapt to more rigid (SRS) and nonrigid treatment sites (regions of the breast or extremities).^29^ The main difference between resolutions is the number of arbitrary points being mapped, and the CRAD Catalyst has two options: standard and SRS‐specific resolutions. Standard resolution is typically used for deformable regions, such as the breast or extremities, while SRS‐specific resolution utilizes a higher resolution to accommodate for a smaller, more rigid surface such as the face for patient's immobilized with open face masks.^29^ It is also important to note that the SGRT system must undergo an SRS‐specific resolution calibration. The system works by using an image registration algorithm to compare the live 3D surface map to a baseline reference image of the patient in original treatment position. Discrepancies are displayed live on the screen for clinicians to refer to.

A Varian Novalis TX linear accelerator (Varian Medical Systems, Palo Alto, CA) with the HD‐120 MLC (2.5 mm leaf width in center of field) was used for radiation delivery and initial verification with cone beam CT (CBCT). The Exactrac Brainlab (Munich, Germany) imaging system was used for pretreatment verification at noncoplanar couch angles.

### 2.2 RTsafe prime phantom

**FIGURE 2:**
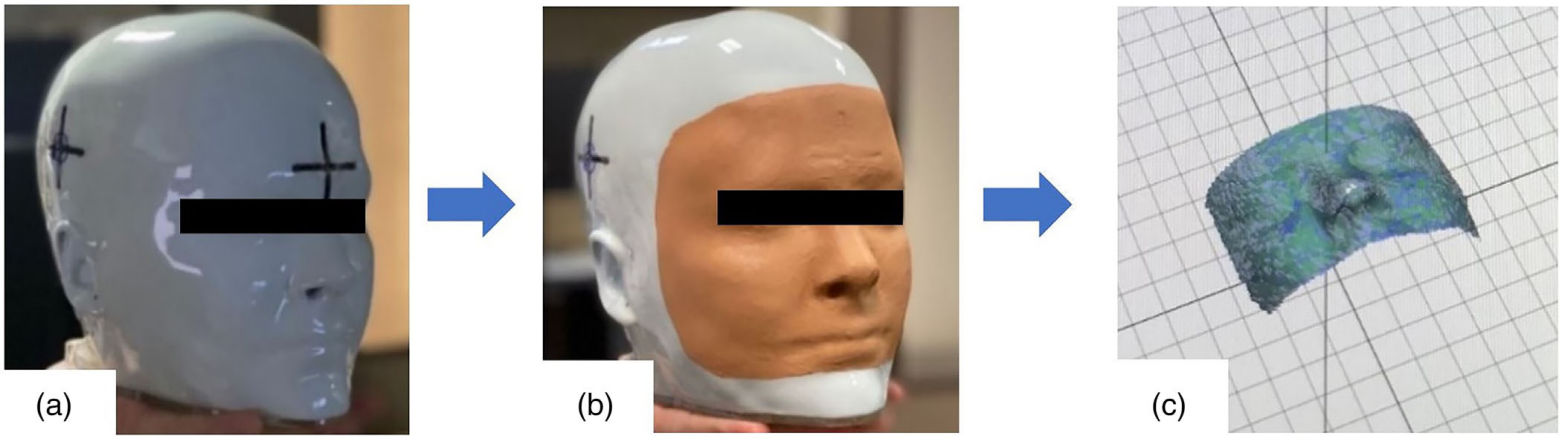
Anthropomorphic (Prime) head phantom (a) without and (b) with makeup foundation applied (c) and the region the surface imaging cameras were able to track with the addition of the makeup foundation. The prime phantom is completely personalized according to patient data and may be recognizable with the addition of the makeup foundation. This head phantom literally represents a patient's internal bone structures for image guidance and external surface, which is important for surface guidance

A 3D‐printed head phantom (Prime phantom, RTsafe PC, Athens, Greece ‐ *see* Figure 2) was used for E2E QA testing. This hollow phantom can be printed with bone‐mimicking materials based on anatomical structures of a past patient, based on CT data or scans, and it is filled with water and a 3D polymer gel dosimeter insert to simulate soft tissue equivalence.^30^ Irradiation of this gel phantom provides the ability to evaluate 3D dose distributions for SRS.^18^, ^31^, ^32^ The custom phantom is designed with a glossy white exterior, which is incompatible with the SGRT cameras. A nude makeup foundation (Stay Matte, Rimmel, London) was applied to the phantom's surface to simulate a skin tone visualizable by the scanner units.

### 2.3 End to end testing

#### 2.3.1 Planning

**FIGURE 3:**
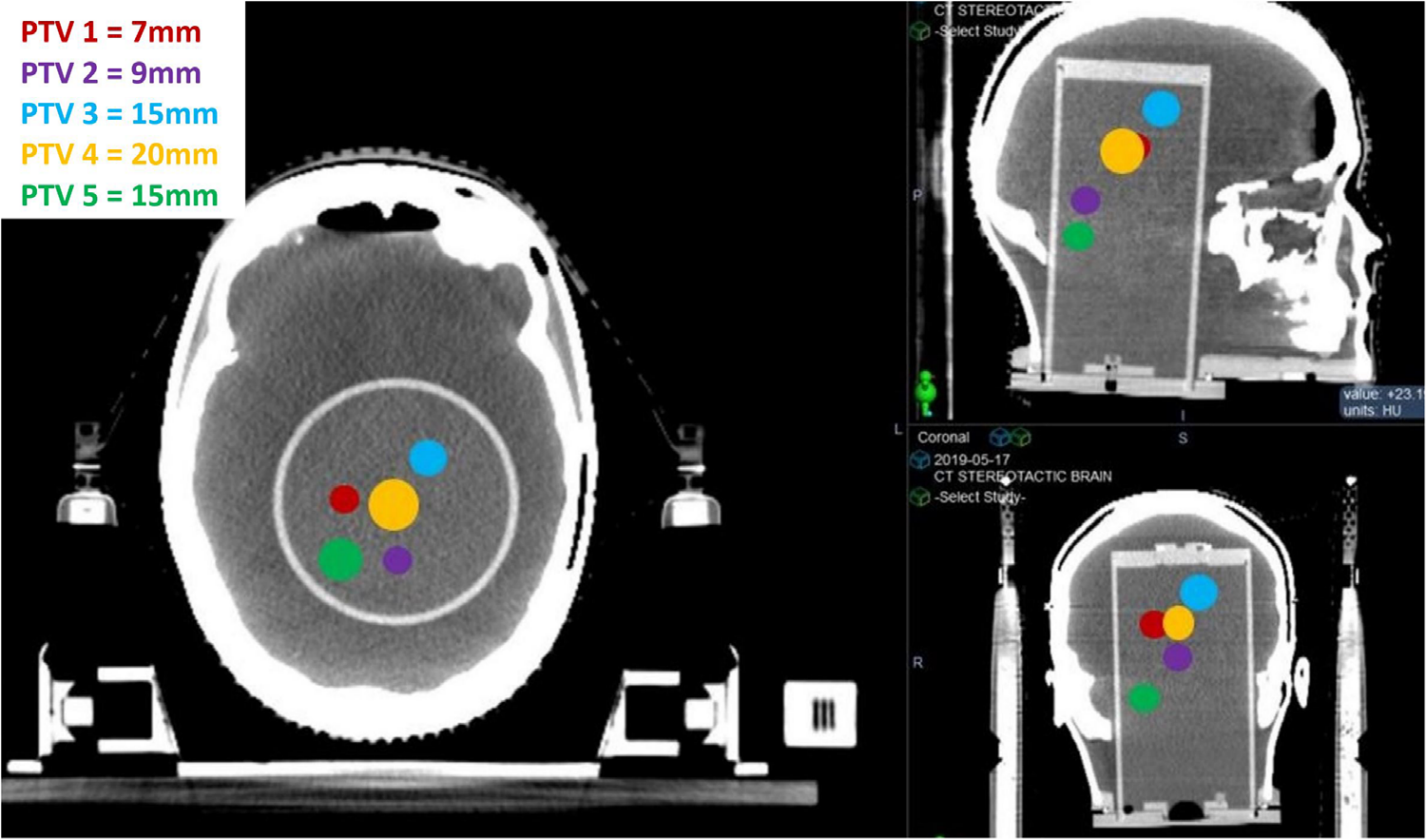
Circles represent approximate geographical locations of planned target volumes (PTVs) targets within the anthropomorphic (Prime) head phantom. All targets fall within the cylindrical gel insert for further analysis of absorbed dose

The radiotherapy plan devised for E2E testing represents one of the most complex SRS treatments in a radiation therapy clinic. Five metastatic brain lesions (see Figure 3) were treated using a single isocenter in 1 fraction. The prescription dose was 8 Gy, and the peaking dose remained under 12 Gy for all five targets. Patient CT and MRI images were imported and fused in the treatment planning system (TPS) (Brainlab Elements Multiple Brain Mets), and these images were segmented to identify targets and organs at risk. These five targets or planned target volumes (PTVs) ranged in diameters from 7 to 20 mm (0.17 to 4.1 cc). Ten arcs were used with five different couch angles. 6 MV photons were used with a dose rate of 1000 MU/min.

#### 2.3.2 Delivery

**FIGURE 4:**
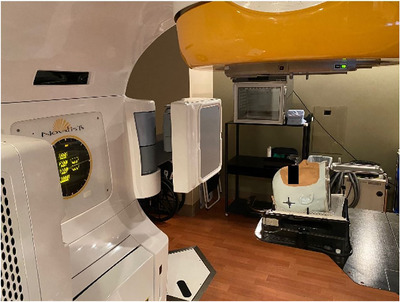
Prime phantom setup below the Varian Novalis TX (Varian Medical Systems, Palo Alto, CA) for end‐to‐end testing

The anatomical head phantom printed for this study was based on a previously treated patient. Two E2E tests were completed using different cylindrical, polymer gel inserts to compare dosimetric accuracy of two isocentric positioning modalities: orthogonal X‐ray imaging and SGRT. One cylindrical gel insert was enclosed in the head phantom, and it was immobilized on the treatment table for setup (see Figure 4).

**FIGURE 5:**
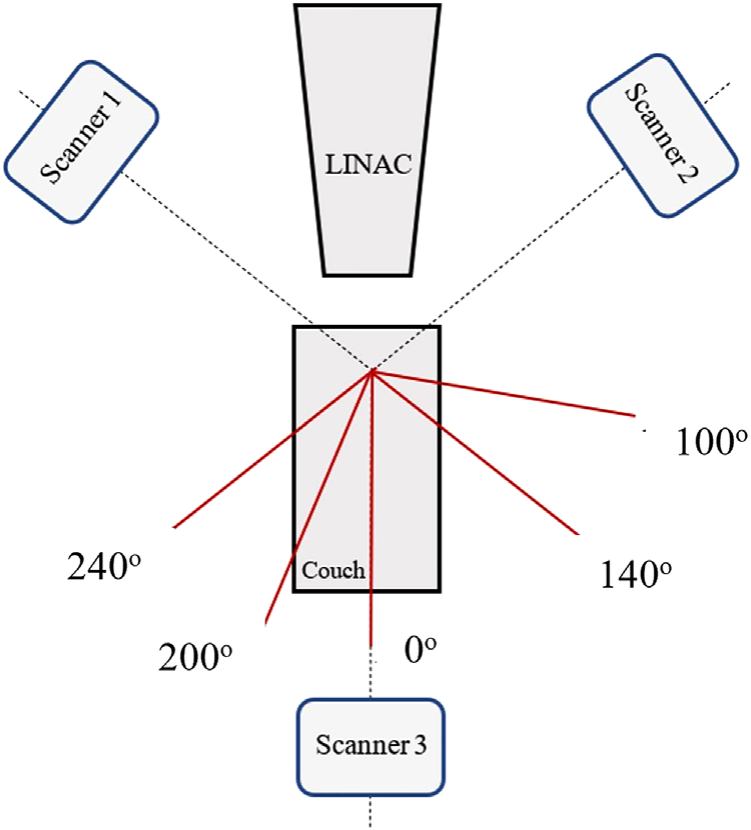
Bird's eye view representation of treatment couch and couch kicks for the treatment plan. Scanner units 1–3 represent the positioning of the surface guided radiation therapy (SGRT) system on the ceiling of the room

**FIGURE 6:**
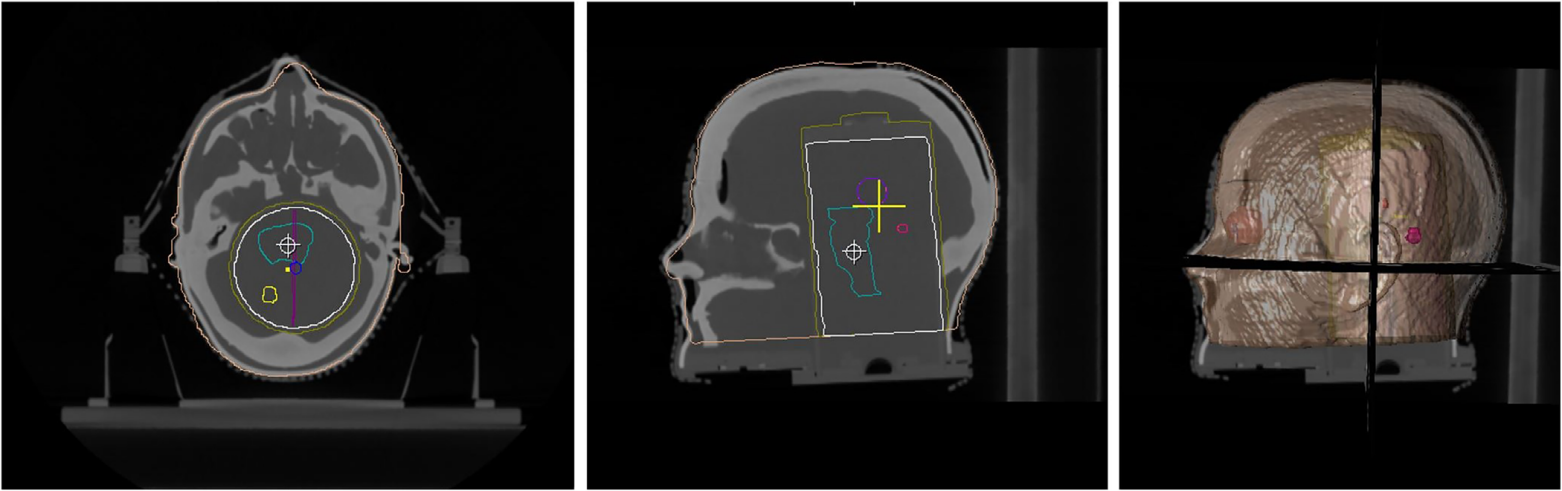
Images demonstrate volume reference data for stereotactic radiosurgery (SRS) treatment plan setup and are captured from Mosaiq (Elekta, Stockholm, Sweden)

The phantom was positioned based on internal anatomy using the standard method of X‐ray imaging (CBCT). Tabletop corrections were made with six degrees of freedom, and the phantom was irradiated. Orthogonal X‐ray imaging was used to verify correct alignment at each couch angle (0^o^, 240^o^, 200^o^, 140^o^, and 100^o^), ultimately representing how this patient was previously treated in the clinic (Figure 5). This gel insert was removed and replaced with a different one for a second E2E test. Figure 6 visually demonstrates internal structure of phantom.

Similar to the first E2E test, the phantom was immobilized for setup, and initial isocenter setup occurred for the linear accelerator table at 0^o^ based on Exactrac and a CBCT to provide a six‐degree, tabletop correction. A reference image was captured by the SGRT system at 0^o^ (CRAD Catalyst HD) to verify correct alignment, based on external anatomy, at treatment couch angles (0^o^, 240^o^, 200^o^, 140^o^, and 100^o^).

Table corrections were made at each couch angle to accommodate position corrections generated by the SGRT system. Adjustments were made so that translational and rotational shift corrections were ≤0.5 mm. In addition, X‐ray images were acquired at each couch angle solely to compare discrepancies in position corrections. This second E2E test represents how a patient would have been treated if they were initially setup using standard CBCT, and position corrections were made at couch angles, according to external anatomy exposed, with an open‐face mask using the SGRT system.

#### 2.3.3 Phantom analysis

**FIGURE 7:**
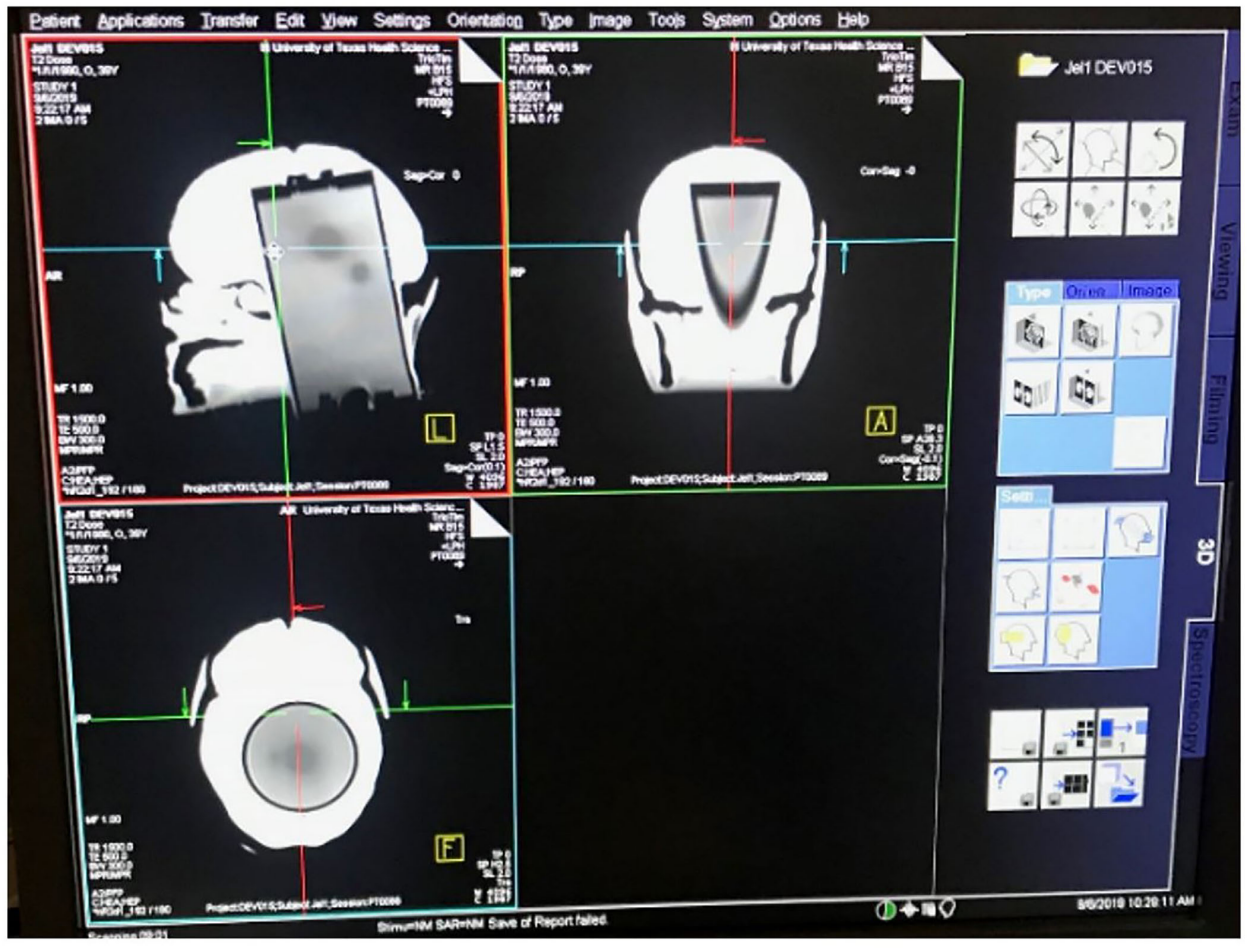
The irradiated Prime phantom scanned on 1.5 T MR unit. The top left image allows you to see how the cylindrical gel insert was placed into the hallow phantom. The remaining space around the gel is water

Forty‐two hours after irradiation, the anthropomorphic Prime phantom was scanned with a head coil on a 1.5 Tesla MRI (Magnetic Resonance Imaging) unit using a 2D multislice, multiecho Proton Density (PD), and T2‐weighted sequences (Figure 7). MR images were fused with the patient CT data set and structure set for further analysis of the resulting dose distributions. The absorbed dose by the gel insert are measured values (3D remote dosimetry service, RTsafe, Athens, Greece) that are compared to the original calculated plan generated in the TPS. This assessment evaluates parameters such as gamma analysis, geometric offset, and dose volume histograms (DVHs).

## 3 RESULTS

### 3.1 Discrepancies between E2E setups

Translational and rotational directions were recorded by the SGRT system (CRAD Catalyst HD) and orthogonal X‐ray imaging system (Exactrac) for the E2E that utilized the SGRT system for positioning at couch rotations. Table 1 displays variation between setup methods with Exactrac shift corrections, SGRT shifts (≤0.5 mm), and discrepancies between 6 degrees of freedom (DOF) table corrections for the second E2E. An increase in discrepancies was apparent when the couch was shifted away from the initial 0^o^. There was <1 mm translational and <0.5° rotational discrepancy at each couch angle, confirming sub‐millimeter accuracies of both modalities.

**TABLE 1:** Translational and rotational shift discrepancies between Exactrac and CRAD system at couch angles

	Exactrac						CRAD						Differences					
Couch angle	Vertical		Longitudinal		Lateral		Vertical		Longitudinal		Lateral		Vertical		Longitudinal		Lateral	
°	mm	°	mm	°	mm	°	mm	°	mm	°	mm	°	mm	°	mm	°	mm	°
0	0.13	0.16	0.14	0.18	−0.10	0.01	−0.10	0.00	0.10	0.00	0.10	0.00	0.23	0.16	0.04	0.18	0.20	0.01
240	−0.49	−0.11	0.76	0.23	0.87	−0.13	−0.10	0.20	0.40	0.10	−0.10	−0.20	0.39	0.31	0.36	0.13	0.97	0.07
200	0.12	0.10	−1.11	0.07	0.84	0.14	0.10	0.30	−0.20	0.00	0.50	0.10	0.02	0.20	0.91	0.07	0.34	0.04
140	−0.29	−0.08	0.41	0.04	−0.47	−0.29	0.30	−0.10	0.10	−0.20	−0.10	0.10	0.59	0.02	0.31	0.24	0.37	0.39
100	−0.38	−0.36	0.75	0.19	−0.42	−0.29	0.20	0.00	0.00	0.20	−0.40	0.10	0.58	0.36	0.75	0.01	0.02	0.39

### 3.2 Motion management

**FIGURE 8:**
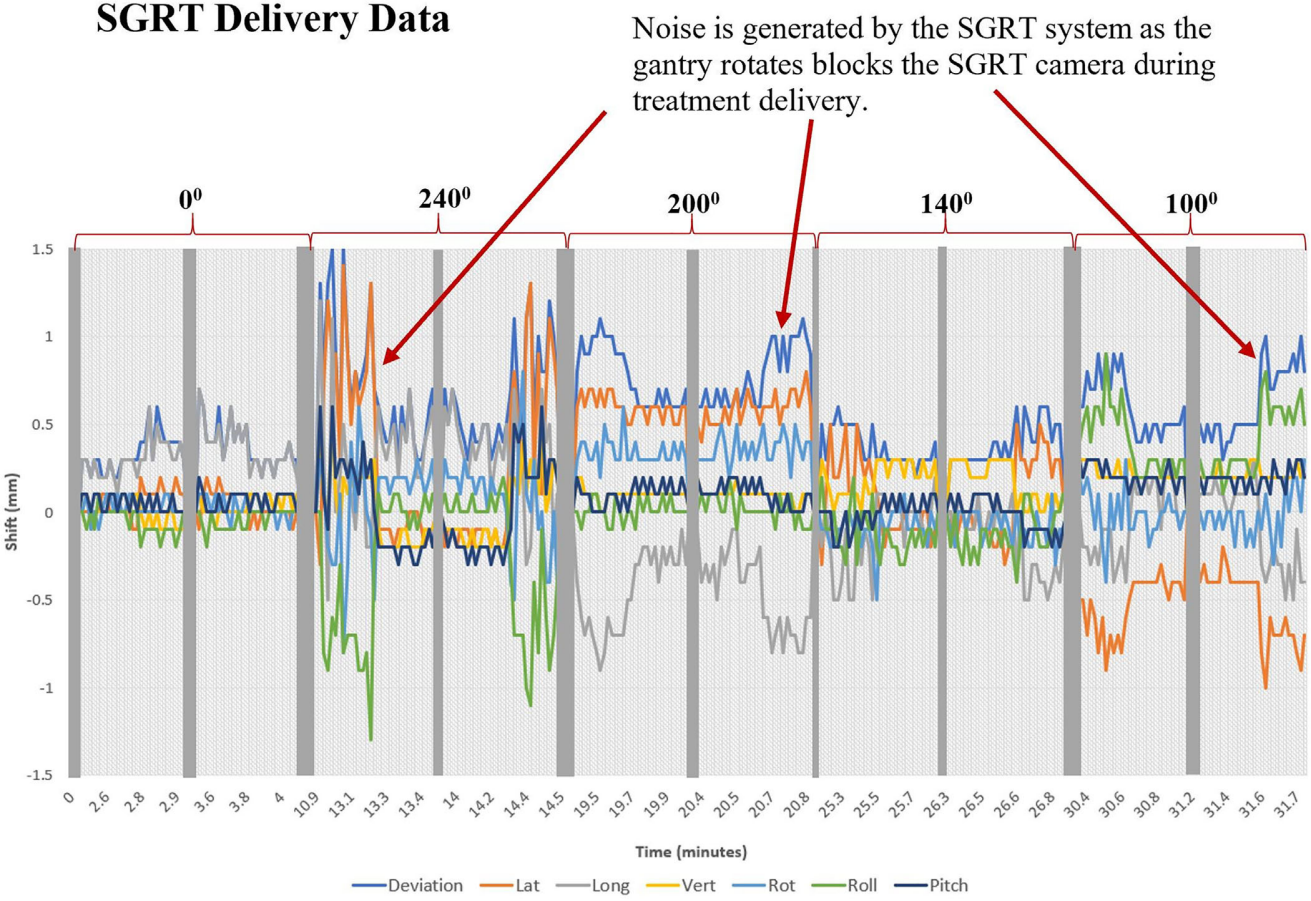
Intrafraction motion data recorded by the surface guided radiation therapy (SGRT) system at each couch angle in the translational and angular directions. Grey bars represent when the SGRT cameras are not tracking positioning. Noise is generated by the SGRT system as the gantry rotates and blocks the SGRT cameras

Intrafraction motion data were recorded by the SGRT system at each couch angle (see Figure 8). Noise was generated by the SGRT system as the gantry rotated and blocked the SGRT cameras. This is significantly amplified at noncoplanar couch angles. Notice that these shifts are decreased after the gantry has moved out of the way.

### 3.3 Prime phantom 3D polymer gel dosimetry

The two smaller targets (PTV 1, 2) showed the lowest discrepancies between positioning modalities for geometric offset. Two of three the largest targets (PTV 3 and 4) provided less geometric offset when positioned according to the SGRT system than with standard orthogonal X‐ray imaging. These same structures had the greatest setup differences between E2E tests (as seen in Table 2).

**TABLE 2:** Planned target volumes (PTVs) diameter, distance from isocenter, and geometric offset based on phantom setup method

	Planned Target Volume (PTV)		Geometric offset (mm)	
	Diameter (mm)	Distance from isocenter (cm)	Exactrac	CRAD
1	7	1.67	1.09	0.99
2	9	1.89	0.75	0.79
3	15	3.82	1.69	0.82
4	20	1.09	1.4	0.85
5	15	4.05	1.25	1.07

**FIGURE 9:**
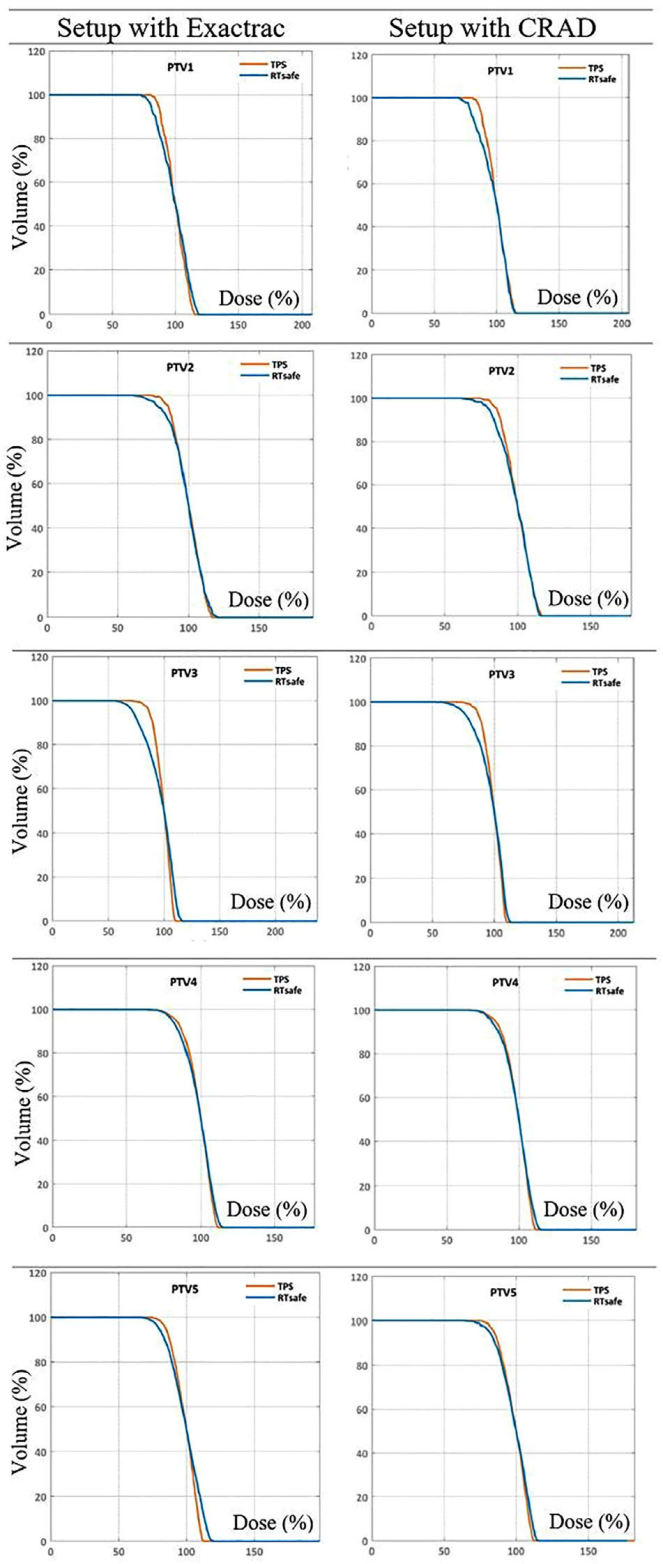
Cumulative dose volume histograms (DVHs) present a comparison between planned (treatment planning system [TPS]) and measured dose distributions (RTsafe) based on positioning methods used for each Planned Target Volume (PTV)

A comparison between planned and measured relative dose distributions were normalized to the *D*_50%_ metric (this is the minimum dose received by at least 50% of the volume) of each structure for cumulative DVHs (see Figure 9). Based on this normalization (100% corresponds to *D*_50%_), the mean dose (*D*_Mean%_) delivered to each target volume and the minimum dose delivered to at least 95% of each target volume (*D*_95%_) were derived from these same DVHs. Table 3 presents dose differences between calculated (TPS) and measured values based on phantom setup method. The target (PTV 3) with the greatest geometric offset also had the largest discrepancy of *D*_95%_. Targets less than 10 mm in size had a *D*_95%_ decrease less than 10% while targets larger than 10 mm (PTV 3, 4, 5) showed a decrease in *D*_95%_ values from 3% to 15% for positioning methods when compared to the TPS.

**TABLE 3:** Dose volume histogram dose metric differences (%) between calculated (treatment planning system [TPS]) and measured values based on phantom setup method

	Dose comparison									
	*D*_Mean%_					*D*_95%_				
PTV	TPS	Exactrac	CRAD	Diff (TPS‐Exactrac)	Diff (TPS‐CRAD)	TPS	Exactrac	CRAD	Diff (TPS‐Exactrac)	Diff (TPS‐CRAD)
1	99.91	99.22	97.73	0.69	2.18	86.58	80.61	78.33	5.97	8.25
2	99.91	99.04	98.57	0.87	1.34	85.33	78.41	80.25	6.92	5.08
3	99.15	96.72	97.32	2.43	1.83	86.68	72.06	75.61	14.62	11.07
4	98.80	98.53	98.69	0.27	0.11	83.65	80.95	81.13	2.70	2.52
5	98.99	99.19	98.86	0.20	0.13	84.79	79.72	81.65	5.07	3.14

Abbreviation: PTV, planned target volume.

**FIGURE 10:**
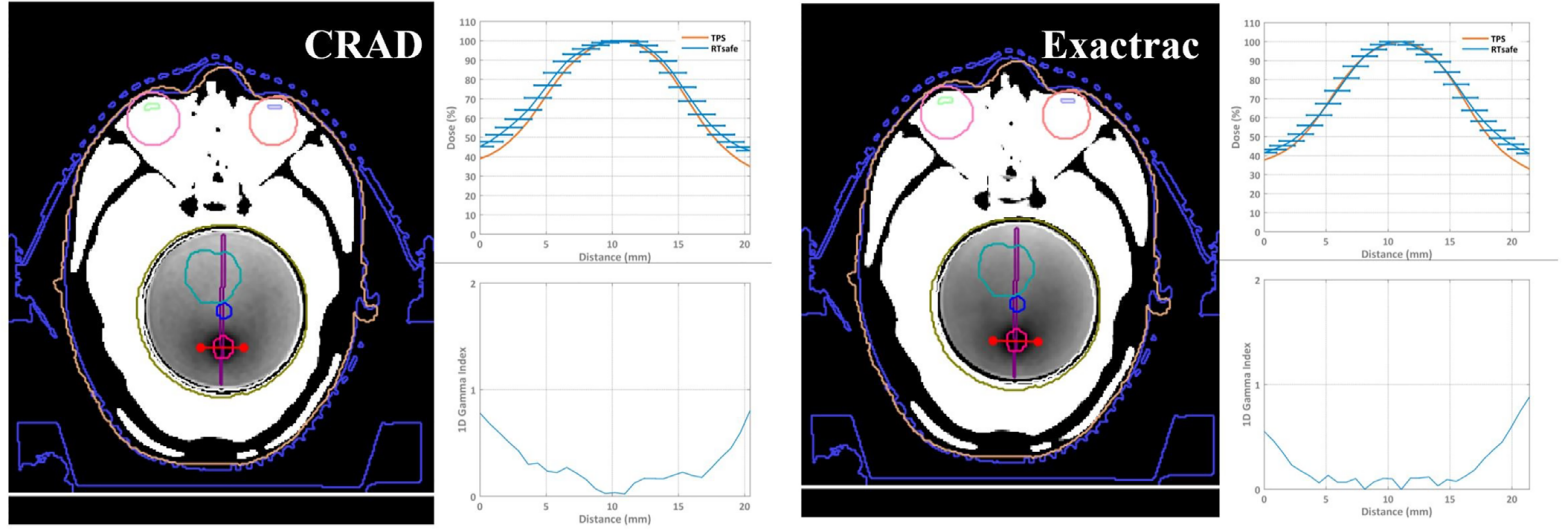
Axial MR image slices and 1D gamma index (2 mm/5%) results for planned target volume (PTV) 2. CRAD had a comparable passing rate to Exactrac. Isocenter positioning methods had the smallest values and smallest discrepancies (0.04 mm) for geometric offset for this target. Darker regions within the phantom correspond to high dose regions. The dose versus distance plots represent a 1D comparison between the calculated (treatment planning system [TPS]) and the measured (MR image analysis by RTsafe) dose distributions. Finally, the 1D gamma index versus distance is given

**FIGURE 11:**
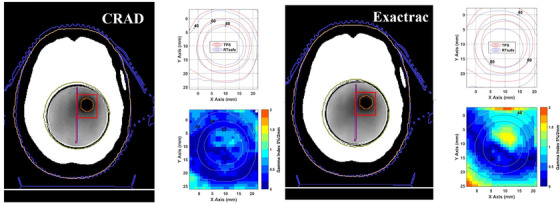
Axial MR image slices and 2D gamma index (2 mm/5% and dose threshold of 1%) results for planned target volumes (PTV) 3. Isocenter positioning methods had the largest discrepancies (0.87 mm) for geometric offset for this target. Darker regions within the phantom correspond to high dose regions. The x versus y plots represent a two‐dimensional comparison between the calculated (treatment planning system [TPS]) and measured (MR image analysis by RTsafe) dose distributions. Finally, the 2D gamma index values are represented according to pixel intensity

Axial MR images and 1D/2D gamma analysis are presented in Figures 10 and 11 for a visual representation of gamma index (GI) analysis. 3D gamma analysis provides a more stringent criterion, and results are summarized in Table 4. For the volumes considered, the GI comparison was performed within a volume of interest that included the target(s) along with an extended region of surrounding soft tissue. PTV structures were evaluated for gamma criterion of 3%/2 mm 5%/2 mm and 5%/1 mm. While 3%/2 mm is a universal action and tolerance limit for patient‐specific QA, these tighter tolerances allow us to detect subtle regional errors in these high‐gradient regions.^33^ The SGRT positioning method performed comparable to standard methods in several ways. For four of five targets, passing rate values exceeded 98% with SGRT‐assisted positioning and 94% with standard methods. The two structures farthest from the isocenter performed with the lowest passing rates with 3%/2‐mm criterion, and this is amplified with the 5%/1‐mm criterion. According to the 5%/1‐mm criterion, surface imaging produced the lowest passing rate for PTV 3 (83.71%) while orthogonal imaging produced an even lower passing rate for PTV 5 (81.84%).

**TABLE 4:** Passing criteria (%) for 3D gamma index values based on phantom setup method

PTV	DD/DTA = 3%/2 mm		DD/DTA = 5%/2 mm		DD/DTA = 5%/1 mm	
	Exactrac	CRAD	Exactrac	CRAD	Exactrac	CRAD
1	93.37	99.33	99.95	99.99	93.14	98.32
2	98.9	98.26	99.47	98.79	91.52	87.27
3	93.1	87.24	97.38	94.57	89.4	83.71
4	97.3	98.53	98.68	99.42	91.05	94.09
5	94.41	98.87	95.57	99.16	81.84	92.76

Abbreviation: PTV, planned target volume.

## 4 DISCUSSION

The results of this study demonstrate the feasibility of using SGRT for positioning at noncoplanar couch angles for mono‐isocentric, multiple‐target SRS using an E2E gel dosimeter. A previous study by Sarkar et al.^34^ demonstrated the feasibility of SGRT for patient shift correction for noncoplanar, multiple‐target SRS treatment comparing AlignRT versus Exactrac. They calculated 3D vector shifts and modalities never differed by more than 1 mm for each couch angle, which is consistent with our results in Table 1. This paper provides a new gel dosimetric comparison of targets not previously published comparing CRAD versus Exactrac.

### 4.1 Gel dosimeters

3D‐printed head phantoms have been used for intracranial radiotherapy applications.^35^, ^36^ More specifically, studies have evaluated the suitability of using 3D‐printed head phantoms with bone mimicking materials for patient‐specific plan verification procedures, and they demonstrate excellent agreement with the actual patient.^18^, ^37^ Gel dosimetry permits evaluation of 3D dose distributions for SRS^18^, ^31^, ^32^ and has been recommended for initial validation of a stereotactic program. However, it is important to note that the equipment needed to produce this phantom may not be easily accessible, cost effective, and could take approximately 9 h to print.^37^

Cumulative DVHs (Figure 9) graphically compare a simulated radiation distribution (or measured values) within a volume of interest that would result from a proposed radiation treatment plan (calculated in TPS).^38^ Any uncertainty from the chain of irradiation could be easily revealed in the experimental DVHs especially for small targets. If an experimentally derived relative cumulative DVH is identical to the TPS‐calculated DVH then there is (1) high spatial accuracy of dose delivery and (2) the 3D shape of the experimental isosurface is identical to the TPS calculated one.

In this study, we see a decrease in D95 values for some targets and most significantly for PTV 3, which has the largest geometric offset when setup with both modalities. Previous studies suggest that rotational and translational inaccuracies as well as increasing distance to isocenter lower target coverage, thus lowering values of D95 in single‐isocenter multitarget SRS.^39^ Roper et al. simulated uniform rotational setup errors for single isocenter multiple target SRS using volumetric modulated arc therapy and observed a decrease in D95 values to ≤60% the prescription dose for a 2° rotation.^39^ Sagawa et al. also observed D95% coverage for single isocenter multiple target SRS, however D95% errors were slightly larger than reported by Roper et al. The union of the multiple PTV targets (PTV_all_) in this study demonstrated a decrease of dose by 10.4%±10.6% under the influence of setup errors and a maximum difference of D95% equal to ‐37.4%.^40^ Differences in coverage may be due to simulating more robust rotational errors or increased sensitivity of errors due to plans generating steeper dose fall off gradients.^41^

A previous study evaluated 3D GIs for single‐isocenter multitarget radiosurgery finding gamma passing rates >90% with 3%/2‐mm tolerances.^42^ We found similar results for five of five PTV structures setup with X‐ray imaging and for four of five PTV structures setup according to SGRT. According to TG 218, Universal Tolerance and Action limits abide by 3%/2 mm (DD/DTA, DD= Dose differences,DTA = Distance to agreement) with ≥95% and ≥90% passing rates, respectively. While DTA is useful for more high gradient regions, tightening these tolerances or lowering DTA allows us to see regional errors.^33^ To further explore these errors, analysis included 5%/1‐mm and 5%/2‐mm tolerances to detect not only dosimetric but also geometrical issues, especially with the criteria 5%/1 mm.

Lee et al.^43^ examined the dependency of SGRT setup differences on the isocenter location for potential bias of targets superficial to the surface. The vertical to isocenter depth indicated a mild bias of increasing setup differences with an increase in distance. In this study, they defined skin‐to‐isocenter distance as a projection of the isocenter to the midplane in the lateral direction and measured the distance to the anterior surface along the lateral midplane. Our plan included a single isocenter for multiple‐targets with a skin‐to‐isocenter distance of 11.7 cm. The shift differences we observed between modalities were lower than the average values observed for translational shifts (2.4 ± 1.7 mm) in this study. We agree with Lee et al. that this dependency is unlikely to be clinically impactful.

### 4.2 SRS positioning

Research recommends minimizing treatment setup errors to provide the most dosimetrically accurate treatment.^15^ An initial patient setup (e.g., laser alignment) is typically followed by X‐ray imaging (e.g., CBCT) prior to radiation delivery to verify positioning of the patient and target.^17^ Modern linacs do not permit CBCT position verification at noncoplanar couch angles,^11^ thus use of additional technology such as the Exactrac X‐ray system (Brainlab AG, Munich, Germany)^44^ or SGRT does enable image verification at noncoplanar couch angles with an added cost of treatment duration. The advantage of SGRT is that it does not require additional imaging dose, and it provides continuous motion management throughout treatment delivery, which may reduce or eliminate need for anesthesia or larger treatment margins, which are typically used to account for motion uncertainty.^45^ It is important to note that treatments utilizing noncoplanar couch angles must also undergo Winston–Lutz tests to verify radiation isocenter.^46^ Clinics implementing SGRT must consider that there could be large shift differences between positioning modalities at extreme couch angles^34^ or that it could generate false positional corrections for patients immobilized in an open face masks.^47^ To reduce surface tracking uncertainties, exposure to the entire face with an open face mask has been recommended.^14^

A 2020 study by Chetvertkov et al. surveyed 568 institutions and reported imaging systems used for position verification as well as steps taken to verify positioning (including extra CBCT or other imaging) during an SRT brain fraction. Note that 59.4% reported using CBCT as their main methodology, 16.5% said 2DkV/portal imaging, 1.2% said they use a surface tracking system, 1.0% reported use of no imaging, and 21.9% (88 institutions) said other. For these 88 other institutions, 46 reported use of BrainLab ExacTrac and/or other imaging, and 16 reported use of a surface tracking system and/or other imaging. When verifying positioning during a single fraction, 53.3% reported that they do not normally acquire an extra image, 35.3% reported that they normally acquire an extra CBCT or other image, and 11.4% (45 institutions) only acquire an extra image under certain circumstances.^48^ Another study that surveyed SGRT use at 439 institutions found that 53.3% reported having SGRT in their clinics with over 10% of them not using it clinically and 36.8% classifying themselves as “expert” users.^49^ Results from these studies powerfully demonstrate how current practices can vary significantly at different institutions, and how the current standard of care for SRS or SRT brain treatments typically ignore intrafraction motion. There is need for national recommendations on use of SGRT,^49^ and as the current standard of care for SRS typically ignores intrafraction motion, we do see an overall benefit for this technique when appropriate tolerances are used.

The surface position tracking data produced in this study generated noise at a few couch angles (some >±1.5 mm), which we believe was due to rotation of the gantry during beam delivery, causing blockage of the SGRT scanner units. When the patient is set up to a new couch angle, we recommend limiting gantry blockage to the camera to minimize noise. During the period of treatment for blocked angles, there is potential for missed intrafraction motion that can be accounted for with repeated X‐ray imaging.^14^ The noise is more clearly due to this study being performed on a phantom; however it may be difficult to distinguish the variations caused by noise from gantry rotation blocking the camera or by actual patient intrafractional motion. Studies have assumed 3D surface tracking data to be degraded or noisy due to the blockage of the system by the gantry,^50^ couch kicks, and effects from in room lighting.^51^ Depending on the optical imaging system being used, it may be possible to turn off the camera when it is being blocked by the gantry during treatment.^23^ Another recommendation to reduce camera blockage was made to change the current configuration of a three‐camera system by replacing it with a four‐camera system (two front cameras instead of one that are angled ±20° from the couch zero position).^52^

If motion is detected at a couch rotation angle using SGRT, it has been recommended to return the couch back to zero position and verify if it was due to patient motion or a false positive due to SGRT couch dependency using another SGRT verification image.^53^ If the patient moved from setup by >1 mm, then they would need to be setup again with CBCT, and the incident would be recorded.^43^ Lee et al. also enlarged the translational threshold to account for couch angle dependency error from SGRT system.

## 5 CONCLUSIONS

The results of this study demonstrate the feasibility of using SGRT for position verification at noncoplanar couch angles for mono‐isocentric, multiple target SRS using end to end gel dosimetry. Setup with SGRT was found to be comparable to using orthogonal X‐ray imaging, demonstrating high accuracy and reproducibility for treatment delivery.

## CONFLICT OF INTEREST

The authors declare that there is no conflict of interest that could be perceived as prejudicing the impartiality of the research reported.

## AUTHOR CONTRIBUTIONS

Daniel Saenz, Evangelos Pappas, Georgios Kalaitzakis, Niko Papanikolaou, and Karl Rasmussen contributed to the design of project while Victoria Bry, Daniel Saenz, Niko Papanikolaou, and Karl Rasmussen contributed to developing conceptual ideas. Victoria Bry and Daniel Saenz carried out the experiment. Victoria Bry, Daniel Saenz, Evangelos Pappas, and Georgios Kalaitzakis contributed to manuscript preparation. Victoria Bry, Evangelos Pappas, and Georgios Kalaitzakis carried out statistical and data analysis. Victoria Bry, Daniel Saenz, Evangelos Pappas, Georgios Kalaitzakis, Niko Papanikolaou, and Karl Rasmussen participated in manuscript editing and review.
